# Fluorescence and Bioluminescence Imaging of Angiogenesis in *Flk1-Nano-lantern* Transgenic Mice

**DOI:** 10.1038/srep46597

**Published:** 2017-04-20

**Authors:** Jun Matsushita, Shigenori Inagaki, Tomomi Nishie, Tomoki Sakasai, Junko Tanaka, Chisato Watanabe, Ken-ichi Mizutani, Yoshihiro Miwa, Ken Matsumoto, Kazuhiro Takara, Hisamichi Naito, Hiroyasu Kidoya, Nobuyuki Takakura, Takeharu Nagai, Satoru Takahashi, Masatsugu Ema

**Affiliations:** 1 Department of Stem Cells and Human Disease Models, Research Center for Animal Life Science, Shiga University of Medical Sciences, Seta, Tsukinowa-cho, Otsu, Shiga 520-2192, Japan; 2 Graduate School of Frontier Biosciences, Osaka University, 1-3 Yamadaoka, Suita, Osaka 565-0871, Japan; 3 Department of Molecular Pharmacology, Faculty of Medicine, University of Tsukuba, Tsukuba 305-8575, Ibaraki, Japan; 4 Laboratory of Neural Differentiation, Graduate School of Brain Science, Doshisha University, 1-3 Tatara Miyakodani, Kyotanabe-shi, Kyoto 610-0394, Japan; 5 Department of Signal Transduction, Research Institute for Microbial Diseases, Osaka University, 3-1 Yamada-oka, Suita, Osaka 565-0871, Japan; 6 Department of Anatomy and Embryology, Faculty of Medicine, University of Tsukuba, Tennodai 1-1-1, Tsukuba, Ibaraki 305-8577, Japan; 7 Center for Tsukuba Advanced Research Alliance, University of Tsukuba, 1-1-1 Tennodai, Tsukuba, Ibaraki 305-8577, Japan; 8 International Institute for Integrative Sleep Medicine, Life Science Center, and Laboratory Animal Resource Center, University of Tsukuba, 1-1-1 Tennodai, Tsukuba, Ibaraki 305-8577, Japan; 9 Precursory Research for Embryonic Science and Technology, Japan Science and Technology Agency, 4-1-8 Honcho Kawaguchi, Saitama 332-0012, Japan

## Abstract

Angiogenesis is important for normal development as well as for tumour growth. However, the molecular and cellular mechanisms underlying angiogenesis are not fully understood, partly because of the lack of a good animal model for imaging. Here, we report the generation of a novel transgenic (Tg) mouse that expresses a bioluminescent reporter protein, *Nano-lantern*, under the control of *Fetal liver kinase 1 (Flk1*). *Flk1-Nano-lantern* BAC Tg mice recapitulated endogenous *Flk1* expression in endothelial cells and lymphatic endothelial cells during development and tumour growth. Importantly, bioluminescence imaging of endothelial cells from the aortic rings of *Flk1-Nano-lantern* BAC Tg mice enabled us to observe endothelial sprouting for 18 hr without any detectable phototoxicity. Furthermore, *Flk1-Nano-lantern* BAC Tg mice achieved time-lapse luminescence imaging of tumour angiogenesis in freely moving mice with implanted tumours. Thus, this transgenic mouse line contributes a unique model to study angiogenesis within both physiological and pathological contexts.

The formation of new blood vessels, termed angiogenesis, is important for embryonic development. The basic steps of angiogenesis include degradation of the extracellular matrix, directed migration of endothelial cells (ECs), EC proliferation, EC tube formation, vessel maturation and stabilization by the recruitment of mural cells, and the deposition of the extracellular matrix. During this process, ECs adopt two distinct cellular types, tip and stalk cells. Endothelial tip cells spearhead new sprouts with dynamic filopodia and determine the direction of migration. Following tip cells, stalk cells extend fewer filopodia and support sprout elongation with their highly proliferative ability^1^. Tip cells anastomose with cells from neighbouring sprouts and form the vascular lumen. Phalanx cells align in a smooth cobblestone monolayer that has tight junctions and contact with mural cells^2^.

A blood vessel network is crucial for tumour progression^3^. Therefore, angiogenesis in tumours is considered to be one of the most important therapeutic targets^4^,^5^. Blood vessels in tumours are highly irregular compared with those in normal organs. Unlike normal vessels, tumour vessels are dilated and tortuous with leaky saccular shapes^6^,^7^. An abnormal endothelial layer is also observed in tumours. Tumour vessels are poorly interconnected with overlapping cells and shunting that compromises blood flow. In addition, it is difficult to distinguish between arteries and veins. These specific phenotypes of tumour vessels lead to poor drug delivery^8^,^9^. Phalanx cells arise in heterozygous *Phd2*-deficient ECs, leading to improvement in treatment with chemotherapy^10^. Tip cells promote the outgrowth of tumour cells in metastatic regions but not the outgrowth of stalk cells^11^.

In the embryo, vascular endothelial growth factor (VEGF)-A is a key factor that regulates angiogenesis^12^. VEGF-A interacts with two receptors, Flt1 and Flk1. Flk1 is known as the primary receptor that regulates most of the endothelial responses such as the proliferation and migration of ECs, vascular permeability, and the selection of tip and stalk cells. Flt1 functions as a decoy receptor because of its weak tyrosine kinase activity^13^. *Flk1* is highly expressed in tip cells. Conversely, *Flt1* expression is more prominent in stalk cells. Furthermore, the morphology of tip and stalk cells is determined by Flk1 signalling^14^.

In tumours, VEGF-A is considered to be one of the most important therapeutic targets. Although recent clinical studies of tumours have indicated that VEGF-A agents show promising benefits, largely negative results have been obtained^15^,^16^,^17^ due to the resistance to Bevacizumab^18^. Therefore, we need to understand the precise molecular mechanisms of VEGF-A/Flk1 signalling during tumour progression.

Because green fluorescent protein (GFP) emits strong and clear green fluorescence, it has been used to monitor gene expression in many studies. However, the fluorescence requires excitation by light that often causes phototoxicity and difficulty in distinguishing GFP fluorescence from the autofluorescence of mammalian tissues. Recently, a chimeric gene encoding the bright fluorescent protein Venus and an enhanced version of Renilla luciferase, called *Nano-lantern*, was developed to overcome these issues^19^. *Nano-lantern* protein emits bioluminescence without light excitation^19^.

Here, we report the generation of a novel transgenic (Tg) mouse that expresses *Nano-lantern* under the control of *Flk1. Flk1-Nano-lantern* BAC Tg mice recapitulate endogenous *Flk1* expression including its expression in the dorsal aorta and micro blood vessels during development and tumour growth. Importantly, bioluminescence imaging of ECs from the aortic rings of *Flk1-Nano-lantern* BAC Tg mice enabled us to observe endothelial sprouting without phototoxicity. Furthermore, *Flk1-Nano-lantern* BAC Tg mice achieved video-rate luminescence imaging of tumour angiogenesis in freely moving mice with implanted tumours. Thus, the EC-specific reporter line will be useful for studying normal and pathological angiogenesis.

## Results

### Generation of Flk1-Nano-lantern BAC transgenic mice

**Figure 1:**
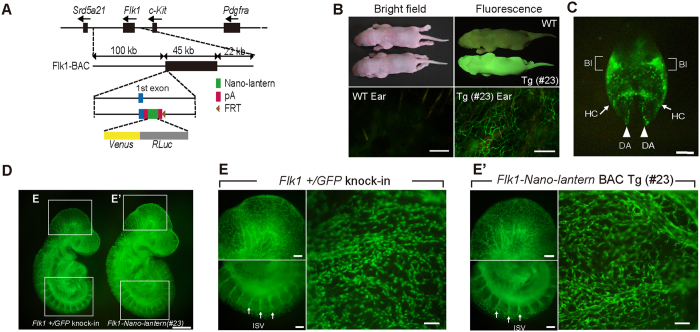
Generation of *Flk1-Nano-lantern* BAC Tg mice. (**A**) Schematic representation of the *Flk1-Nano-lantern* BAC transgene used in this study. The *Flk1* BAC clone (RP24–125B24) was used to drive *Nano-lantern*, a chimeric gene encoding the bright fluorescent protein Venus and an enhanced version of Renilla luciferase (RLuc). (**B**) Gross appearance of *Flk1-Nano-lantern* BAC Tg mice. Bright field and Venus fluorescence images (top panel) of newborns and an adult ear are shown. (**C**) Venus expression in the blood vessels of an 8.5 dpc embryo. DA: dorsal aorta; BI: blood island; HC: heart crescent. (**D**) Fluorescence intensity in an *Flk1*+/*GFP* knock-in embryo and an *Flk1-Nano-lantern* BAC Tg embryo (#23). (**E**) (E′) Venus expression in the blood vessels of a 9.5 dpc *Flk1*+/*GFP* knock-in embryo (**E**) and *Flk1-Nano-lantern* BAC Tg embryo (#23) (E′). Arrows indicate ISV. ISV: intersomitic vessel. Scale bars: 100 m (B), 300 m (C), 500 m (**D**), and 200 m (**E**).

Previously, we generated *Flk1-GFP* BAC Tg mice that are useful for monitoring *Flk1* gene activity during development and in adults^20^,^21^. However, when *Flk1-GFP* BAC Tg mice were used for live imaging of ECs^22^, the ECs that sprouted from an aortic ring of adults underwent apoptosis gradually because of phototoxicity, so the model was unsuitable for long-term (>6 hr) live imaging (K. M. unpublished observation). Recently, the Nano-lantern bioluminescent protein was developed so that Venus luminesces in yellowish-green light when luciferase catalyses a substrate such as coelenterazine-h^19^, achieving live imaging without phototoxicity. Therefore, *Nano-lantern* was introduced into the *Flk1* BAC clone (Fig. 1A) and the resulting *Nano-lantern*-carrying BAC was injected into pronuclei to create *Flk1-Nano-lantern* BAC Tg mice. Five of the eight lines showed clear Venus fluorescence in the adult ears. *Flk1-Nano-lantern* BAC Tg mice were genotyped routinely by inspection of Venus fluorescence in neonatal skin or the adult ear (Fig. 1B, Supplementary Fig. 1A). When embryonic expression of Venus in *Flk1-Nano-lantern* BAC Tg mice was analysed, Venus expression was localized in blood vessels including the dorsal aortae and the vascular plexus of the yolk sac (Fig. 1C).

### Expression of Venus from Flk1-Nano-lantern during development

**Figure 2:**
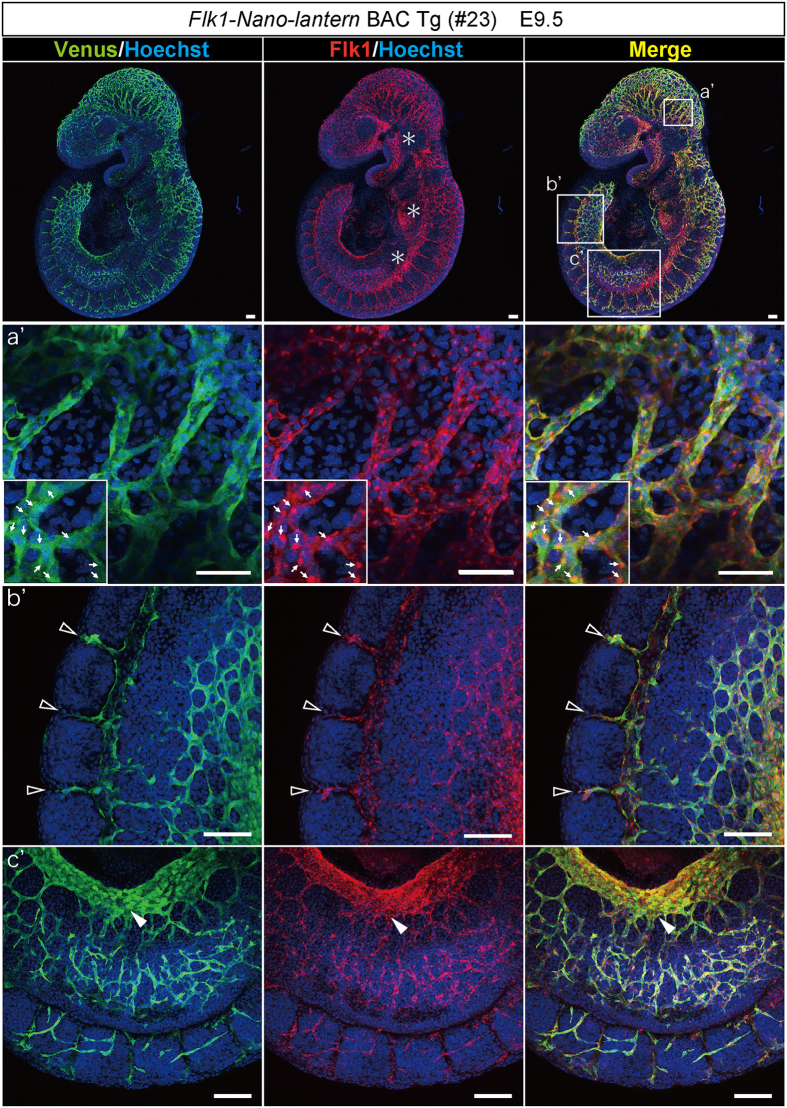
Co-expression of endogenous Flk1 and *Nano-lantern* driven by the *Flk1* regulatory sequence in vascular ECs. Co-expression of Venus and the endogenous *Flk1* protein in *Flk1-Nano-lantern* BAC Tg embryos at 9.5 dpc. Tg embryos were subjected to immunohistochemical analysis with anti-GFP and anti-Flk1 antibodies. *Indicates strong autofluorescence caused by circulating blood cells. Circulating blood cells localized in blood vessels are also shown by arrows. Open arrowheads and a closed arrowhead indicate intersomitic vessels and the vitelline vein, respectively. Scale bars: 100 μm.

**Figure 3:**
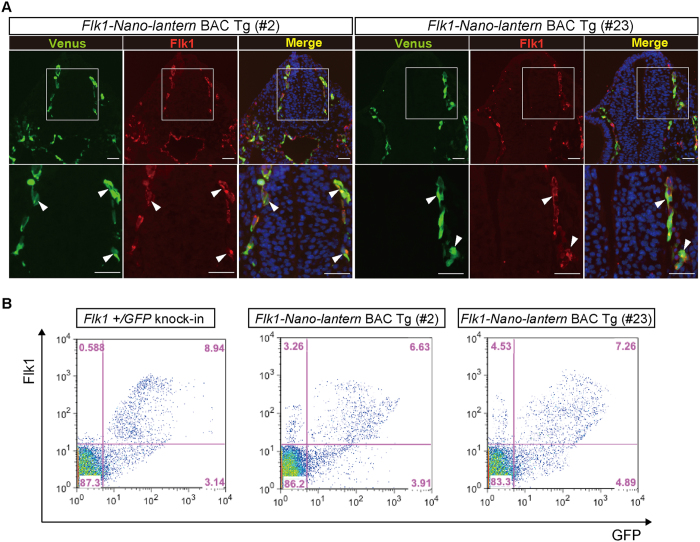
Faithful expression of *Nano-lantern* driven by the *Flk1* regulatory sequence in vascular ECs. Venus expression in *Flk1-Nano-lantern* BAC Tg embryos at 9.5 dpc. (**A**) Co-expression of Venus and the endogenous Flk1 protein. Transverse sections posterior to the heart of *Flk1-Nano-lantern* BAC Tg embryos (#2 and #23) were subjected to immunohistochemical analysis with anti-GFP and anti-Flk1 antibodies to further compare the distribution of their expression. Arrowheads indicate ECs with co-expression of Venus and Flk1. Scale bars: 50 μm. (**B**) Flow cytometric analysis of *Flk1-Nano-lantern* BAC Tg embryos at 9.5 dpc (#2 and #23). Single cells prepared from *Flk1-Nano-lantern* BAC Tg embryos was incubated with PE-labelled anti-Flk1 antibody and flow cytometry was performed as described in Materials and Methods.

To examine whether *Flk1-Nano-lantern* BAC Tg mice are useful for fluorescence imaging, we compared the fluorescent intensity of Venus in ECs of *Flk1-Nano-lantern* BAC Tg mice with that of *Flk1*+/*GFP* knock-in mice^23^ because *Flk1*+/*GFP* knock-in mice have been used to monitor endogenous *Flk1* protein and in other experiments^23^,^24^,^25^ including live imaging^24^. The fluorescence intensity of Venus in the blood vessels of *Flk1-Nano-lantern* BAC Tg mice at embryonic day (E) 9.5 was comparable with that of *Flk1*+/*GFP* knock-in mice (Fig. 1D, Supplementary Fig. 1B). Higher magnification of *Flk1-Nano-lantern* BAC Tg embryos revealed Venus fluorescence in blood vessels at a comparable level as that in *Flk1*+/*GFP* knock-in mice (Fig. 1E and E′, Supplementary Fig. 1C). To compare Venus expression with endogenous *Flk1* protein expression, we performed immunohistochemistry at E9.5 and found that Venus expression overlapped well with *Flk1* protein expression in ECs including in the dorsal aorta, vitelline vein and micro capillaries (Fig. 2, Supplementary Fig. 2). Furthermore, immunohistochemistry on tissue sections confirmed the overlapping expression of endogenous Flk1 and Venus (Fig. 3A). Flow cytometric analysis of E9.5 foetuses also clearly indicated the expression of Venus in ECs of *Flk1-Nano-lantern* BAC Tg mice, which overlapped with endogenous *Flk1* at the cellular level and was consistent with that in *Flk1*+/*GFP* knock-in mice (Fig. 3B).

**Figure 4:**
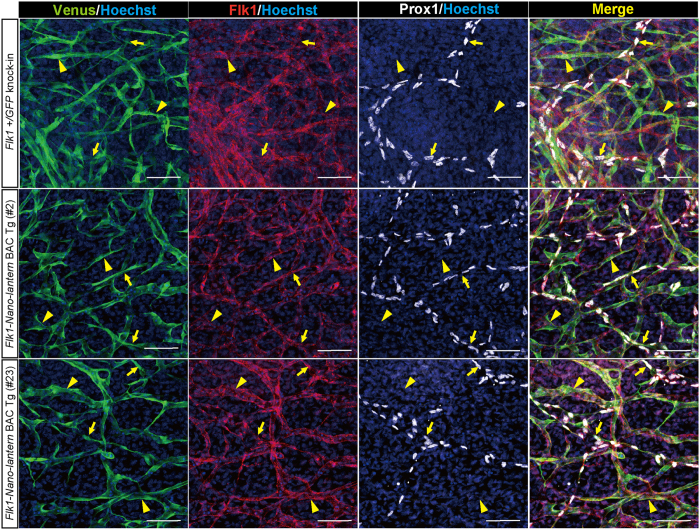
Expression of *Flk1*-*Nano-lantern* in lymphatic ECs. Immunohistochemical analysis of the back skin of *Flk1-Nano-lantern* BAC Tg mice with anti-GFP, -Flk1 and –Prox1 antibodies. Arrows and arrowheads indicate Prox1-positive lymphatic ECs and Prox1-negative vascular ECs, respectively. Scale bars: 50 μm

**Figure 5:**
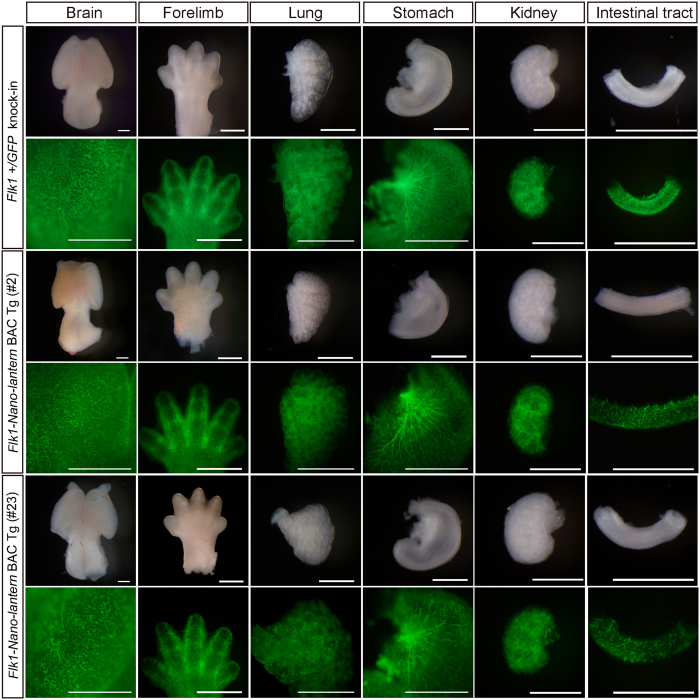
Expression of Venus in various organs from *Flk1-Nano-lantern* BAC Tg mice. Organs from *Flk1*+/*GFP* mice *and Flk1-Nano-lantern* BAC Tg mice (#2 and #23) were dissected out and subjected to imaging with a Leica FLIII. Scale bars: 1 mm.

Previous studies demonstrate the expression of *Flk1* in lymphatic ECs, which has important functions during lymphangiogenesis^26^. To examine Venus expression in lymphatic vessels, the back skin of a *Flk1-Nano-lantern* BAC Tg foetus at E13.5 was removed and subjected to immunohistochemistry for lymphatic vessel markers, Prox1 (Prospero homeobox 1) and LYVE-1 (lymphatic vessel endothelial hyaluronan receptor 1). We found that Prox1 and LYVE-1-positive lymphatic ECs integrated into tubular structures were positive for Venus, indicating that Venus expression driven by *Flk1-Nano-lantern* BAC recapitulates the endogenous expression of Flk1 in lymphatic ECs (Fig. 4, Supplementary Fig. 3). We also examined Venus expression in various organs of *Flk1-Nano-lantern* BAC Tg mice at E13.5. Overall, most organs from *Flk1-Nano-lantern* BAC Tg mice exhibited a similar intensity and pattern of luminescence as those in *Flk1*+/*GFP* knock-in mice (Fig. 5).

### Luminescence imaging of ECs in aortic rings from Flk1-Nano-lantern BAC Tg mice

**Figure 6:**
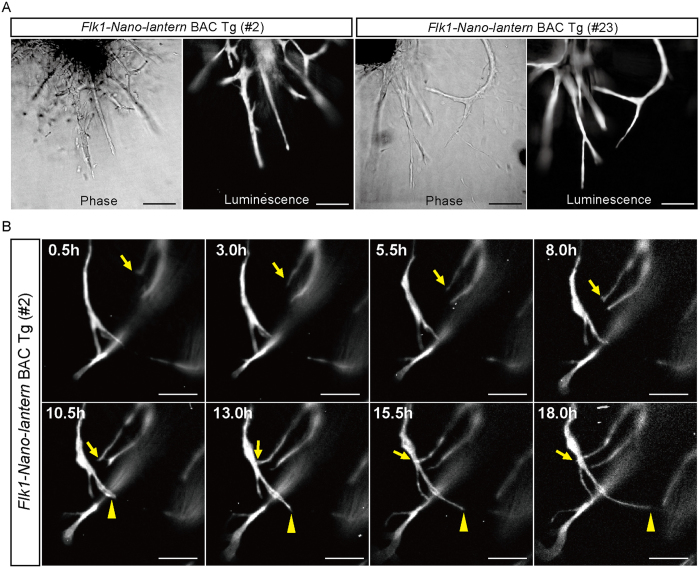
Luminescence imaging of ECs in aortic ECs of *Flk1-Nano-lantern* BAC Tg mice. (**A**) Detection of luminescence in endothelial cells in the aortic rings from *Flk1*+/*GFP* mice and *Flk1-Nano-lantern* BAC Tg mice. The aortic rings were isolated from *Flk1*+/*GFP* mice and *Flk1-Nano-lantern* BAC Tg mice (#2 and #23) and cultured in the presence of VEGF-A. Luminescence was detected after the addition of coelenterazine-h to the media. Scale bars: 100 μm. (**B**) Time-lapse luminescence imaging of sprouting ECs. Luminescence was detected every 30 min and recorded over an 18 hr period. Arrows and arrowheads indicate sprouting ECs. Scale bars: 10 μm.

The aortic ring assay is the standard method to evaluate angiogenesis *in vitro* by measuring the number and length of sprouting blood vessels from an aortic ring^27^. Aortic rings were isolated from *Flk1-Nano-lantern* BAC Tg mice and *Flk1*+/*GFP* mice and were cultured in the presence of VEGF-A for 1 week so that the aortic rings extended sprouts in a VEGF-dependent manner. Immunohistochemistry with anti-GFP and anti-Flk1 antibodies clearly showed that the ECs that sprouted from the aortic rings of *Flk1-Nano-lantern* BAC Tg mice exhibited a similar level and pattern of fluorescence as GFP in *Flk1*+/*GFP* knock-in mice (Supplementary Fig. 4). This result demonstrated that *Flk1-Nano-lantern* BAC Tg animals are useful for visualizing *Flk1*-expressing ECs *in vitro*. However, the fluorescence requires excitation by light that often causes phototoxicity and difficulty in distinguishing GFP fluorescence from the autofluorescence of mammalian tissues. In fact, *Flk1-GFP* BAC Tg mice were unsuitable for long-term (>6 hr) live imaging due to phototoxicity (K. M. unpublished observation). When the aortic rings from *Flk1-Nano-lantern* BAC Tg mice were subjected to bioluminescence imaging according to Saito *et al*.^19^, a bright bioluminescence was detected (Fig. 6A). Furthermore, by capturing the luminescence every 30 min over a period of 18 hr, the observation of EC sprouting was achieved without phototoxicity (Fig. 6B, Supplementary Movie S1), showing a clear advantage over *Flk1-GFP* BAC Tg mice.

### Bioluminescence imaging of tumour blood vessels in Flk1-Nano-lantern BAC Tg mice

**Figure 7:**
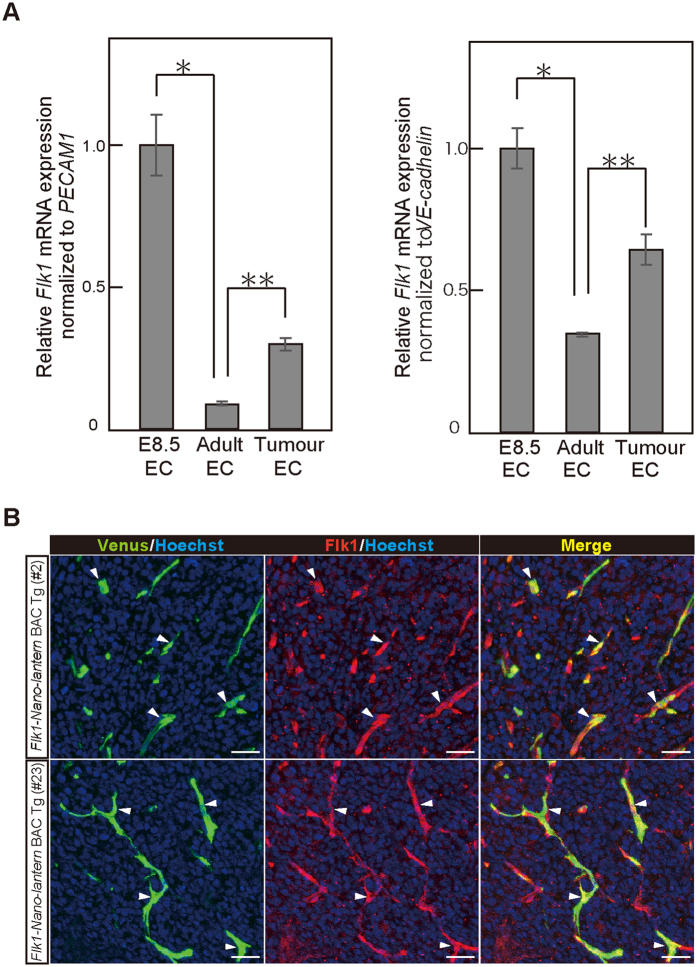
Bioluminescence imaging of tumour blood vessels in *Flk1-Nano-lantern* BAC Tg mice. Relative *Flk1* gene expressions in ECs from embryos at E8.5 (E8.5 EC), adult skin (**A**) Adult EC), and LLC tumours (Tumour EC) normalized by PECAM1 (platelet endothelial cell adhesion molecule-1) (left) and VE-cadherin (right). (**B**) Immunohistochemical analysis of LLC tumours in Flk1-Nano-lantern BAC Tg mice (#2 and #23) with anti-Flk1 and anti-GFP antibodies. Arrowheads indicate ECs with co-expression of Venus and Flk1. Scale bars: 100 μm. LLC cells were implanted into the backs of Fl*k1-Nano-lantern* BAC Tg mice and maintained for 8 days.

*Flk1* is expressed abundantly in the ECs of developing embryos but decreases to a low level in adult tissues^28^,^29^. However, it is re-activated in the neovasculature associated with tumour formation^30^. Accordingly, RT-qPCR analysis showed that *Flk1* mRNA was expressed abundantly in ECs from embryos at E8.5 and down-regulated in CD31-positive/CD45-negative ECs isolated from adult skin (Fig. 7A). Further RT-qPCR analysis showed that Flk1 mRNA was up-regulated by three-fold in CD31-positive/CD45-negative ECs of LLC tumours 8 days after implantation (Fig. 7A). Consistent with the RT-qPCR data, immunohistochemical analysis with anti-Flk1 antibody clearly demonstrated that Venus and Flk1 expressions are well-overlapped in the tumour endothelial cells (Fig. 7B).

**Figure 8:**
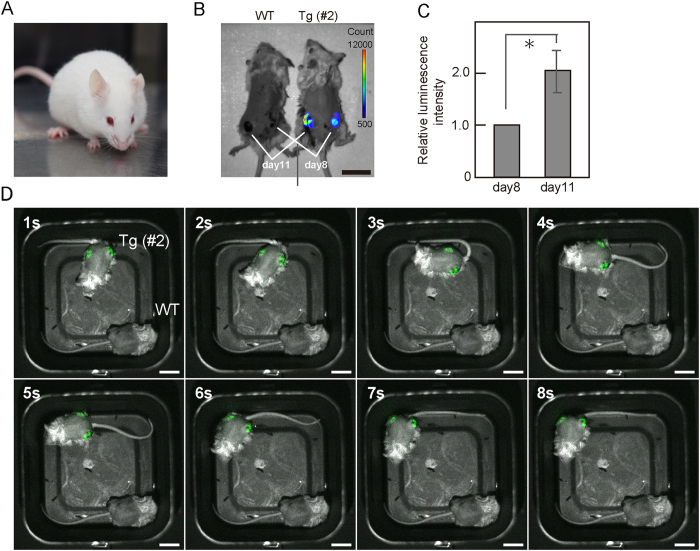
Bioluminescence imaging of tumour ECs in *Flk1-Nano-lantern* BAC Tg mice. (**A**) Coat colour of *Flk1-Nano-lantern* BAC Tg:: B6 (Cg)-Tyr^c−2J^ mice. (**B**) Representative luminescence image of *Flk1-Nano-lantern* BAC Tg mice (#2) with LLC tumours (day 8 and day 11) at 30 s exposure. Scale bar, 2 cm. (**C**) Relative luminescence intensity of the tumours. Luminescence from the tumours on day 8 and day 11 was measured, and the relative value of the luminescence of the tumour on day 11 to that on day 8 was calculated. Asterisks indicate statistical significance. *P < 0.05. (**D**) Consecutive frames of video-rate luminescence images of *Flk1-Nano-lantern* BAC mice with LLC tumours. The luminescence signal every 1 s is shown in green. Scale bar, 2 cm.

To detect bioluminescence from *Flk1-Nano-lantern* BAC Tg mice with a C57BL/6 background, the mice were back-crossed to the C57BL/6 albino (B6 (Cg)-Tyr^c−2J^) strain (Fig. 8A). LLC cells were implanted into the backs of *Flk1-Nano-lantern* BAC Tg mice at two different time points, and *Flk1-Nano-lantern* BAC Tg mice showed two tumours of different sizes from the cells implanted 8 days and 11 days before (Fig. 8B). When coelenterazine-h was administered into the tumour as described in Materials and Methods, we detected a rapid up-regulation of bioluminescence in the tumours of *Flk1-Nano-lantern* BAC Tg mice (Fig. 8B), but no detectable background in WT mice. The intensity of the bioluminescence in the tumour on day 11 was stronger than that on day 8 (Fig. 8C). Furthermore, owing to the intense bioluminescence, we were able to observe luminescence from the tumour ECs in freely moving mice at a video-rate (Fig. 8C). Taken together, these results demonstrated that *Flk1-Nano-lantern* BAC Tg mice are a valuable transgenic animal for the non-invasive detection of pathological angiogenesis, such as tumour angiogenesis, in live mice.

## Discussion

Similar to *Flk1*+/*GFP* knock-in mice, we have shown that the *Flk1-Nano-lantern* BAC Tg mice generated in this study recapitulate endogenous *Flk1* expression in blood vessels during development. *Flk1-Nano-lantern* BAC Tg mice also recapitulated *Flk1* expression in tumour ECs. Previously, Kappel and colleagues identified enhancer activity in the first intron of the *Flk1* gene to direct *Flk1* expression in ECs during development^31^. This enhancer was successfully used to drive mCherry expression in ECs by other researchers. However, deletion of this enhancer has no significant effect on *Flk1* expression in ECs at 9.5 days post-coitis (dpc) or mesodermal progenitors at 7.5 dpc^23^. These results suggest that an unidentified enhancer directs *Flk1* expression in ECs and mesodermal cells. BAC Tg mice and a comparative genome approach showed that the DMME (distal multipotent mesoderm enhancer) located 15 kb upstream of the *Flk1* gene directs Flk1 expression in mesodermal cells during gastrulation and in neural cells of the retina^21^,^22^,^23^,^24^,^25^,^26^,^27^,^28^,^29^. Therefore, the *Flk1-Nano-lantern* BAC Tg mice generated in this study will be useful for recapitulating endogenous *Flk1* expression in ECs as well as neural and mesodermal cells during development.

Flk1 was expressed not only in vascular ECs but also in lymphatic ECs, which regulates lymphatic vessel sprouting by forming heterodimers with Flt4^32^. Albuquerque and colleagues reported that a splice variant of the gene encoding Flk1, which was called soluble Flk1 (sFlk1), regulates lymphangiogenesis by blocking VEGF-C functions^26^. sFlk1 enhances corneal allograft survival and suppresses lymphangioma cell proliferation. These results indicate that sFlk1 might become a therapeutic agent for lymphatic diseases such as lymphatic vascular malformations and tumour lymphangiogenesis. Because our *Flk1-Nano-lantern* BAC Tg mice recapitulate lymphatic Flk1 expression, they would be useful for visualizing *Flk1* expression non-invasively during lymphangiogenesis associated with human diseases.

*Nano-lantern*-expressing cells have been clearly visualized by their bioluminescence [18]. Consistent with that previous report, a bright bioluminescence was detected in ECs of the aortic rings from *Flk1-Nano-lantern* BAC Tg mice, and time-lapse imaging of EC sprouting over 18 hr was achieved without phototoxicity (Fig. 6, Supplementary Movie S1), showing a clear advantage over *Flk1-GFP* BAC Tg mice.

Flk1 is a major mediator of tumour angiogenesis. Therefore, targeting its pathway has been considered an integral component of cancer treatment. Bevacizumab (Avastin), a humanized anti-VEGF antibody, was the first angiogenic inhibitor for cancer patients^15^. Sunitinib (Sutent) inhibits platelet-derived growth factor receptors and VEGF receptors as a multiple receptor tyrosine kinase inhibitor, which was approved by the U.S. Food and Drug Administration for the treatment of renal cell carcinoma and imatinib-resistant gastrointestinal stromal tumours^33^. The multikinase inhibitor sorafenib (Nexavar) is considered a useful drug for the treatment of renal cancer and hepatocellular carcinoma^34^,^35^. Because we have shown that *Flk1-Nano-lantern* BAC Tg mice can be used for bioluminescence imaging of tumour vessels even in freely moving mice, the detection sensitivity for the bioluminescence is very high and this Tg mouse line would be applicable for screening anti-tumour angiogenesis drugs.

## Materials and Methods

### Construction of the Flk1-Nano-lantern BAC transgene

A BAC clone (RP24–125B24) encompassing the *Flk1* locus was modified using the RED/ET recombination method (Gene Bridges, Heidelberg, Germany)^20^. A DNA fragment encoding *Nano-lantern-SV40 polyA*^19^ was ligated to a DNA fragment encoding an FRT PGK-gb2 neo expression cassette that comprises a prokaryotic promoter and neomycin-resistance gene flanked by FRT sequences (Gene Bridges). Homology arms for the first exon of *Flk1* were amplified by two pairs of primers as follows: 5′arm-1: 5′-cgaagagagttctgcacttgcaggc-3′, 5′arm-2: 5′-ggcacagactggttctccgtccctg-3′, 3′arm-1: 5′-catcggttggagcgtgtcctgcggag-3′, and 3′arm-2: 5′-gagttatttagtttaatacacctgg-3′. Then, the arms were ligated to both ends of the reporter cassette. The neo cassette was excised by inducing FLPe expression after introduction of the FLPe expression plasmid (Gene Bridges). Recombination of BAC clones was confirmed by PCR analysis. *Flk1-Nano-lantern* BAC DNA was prepared using a Nucleobond Xtra BAC column (Macherey-Nagel, Düren, Germany) and then linearized by Pl-SceI digestion. The BAC DNA was then subjected to pulse field electrophoresis.

### Mice

Experimental procedures were approved by the Animal Care and Use Committee of Shiga University of Medical Science and methods were carried out in accordance with the approved guideline (Approval number: 13–105). *Flk1-Nano-lantern* BAC Tg DNA was injected into the pronuclei of fertilized eggs collected from C57BL6/J females. The injected eggs were transplanted into pseudo-pregnant ICR females (SLC Inc., Shizuoka, Japan). *Flk1-Nano-lantern* BAC Tg animals were maintained by mating with C57BL6/J mice and genotyped by PCR using the following primers: GFP-S: 5′-AGCAAGGGCGAGGAGCTGTTCACC-3′ and GFP-AS: 5′-TGCCGTCGTCCTTGAAGAAGATG-3′. Fluorescent signals in the skin and ears were used to discriminate *Flk1-Nano-lantern* BAC Tg and wild-type (WT) mice. Fluorescence images of postnatal mice were taken by a Nikon D3300 camera with an AF-S DX NIKKOR 18–55 mm f/3.5–5.6 G VR II lens (Nikon, Tokyo, Japan). A filter (Marumi Cross Screen, Tokyo, Japan) was used to detect GFP fluorescence under an Ex FlashLight ExF-MxB (BioTools, Jupiter, FL, USA). For the detection of bioluminescence, *Flk1-Nano-lantern* BAC Tg animals on a C57BL6/J background were back-crossed by mating with B6 (Cg)-Tyr^c−2J^ mice (The Jackson Laboratory, Bar Harbor, ME, USA).

### Immunohistochemistry

Embryos and tumours were dissected in cold PBS, fixed in 4% paraformaldehyde overnight, and mounted in OCT (Surgipath FSC 22 Blue Frozen section compound; Leica, Wetzlar, Germany) as described previously^36^. Tissue blocks were sectioned using a cryostat (CM1860 UV; Leica). The sections from the embryos were incubated with a rat monoclonal antibody against mouse Flk1 (clone AVAS12, BD Pharmingen, Franklin Lakes, NJ, USA) and an Alexa Fluor 488-conjugated rabbit polyclonal anti-GFP antibody (A21311; Life Technology, Carlsbad, CA, USA) overnight at 4 °C. Bound primary antibodies were detected with secondary Cy5-conjugated donkey anti-rat IgG (712–175–153; Jackson Immuno Research, West Grove, PA, USA). The sections from the LLC tumours were incubated with a rabbit monoclonal antibody against mouse Flk1 (clone 55B11, Cell Signaling, Danvers, MA, USA) and subsequently Alexa Fluor 594-conjugated donkey polyclonal anti-rabbit IgG antibody (Life Technology). Then, Alexa Fluor 488-conjugated rabbit polyclonal anti-GFP antibody (Life Technology) was used to detect GFP overnight at 4 °C. Nuclei were stained with 5 g/ml Hoechst 33342 (Invitrogen, Carlsbad, CA, USA).

Whole mount immunohistochemistry on the embryos was performed as described previously^21^. Embryos were dissected and fixed in 4% paraformaldehyde overnight. After washing with PBS, the embryos were treated with a blocking solution (2% dry skim milk and 0.1% Tween 20 in PBS) for 1 hr and then incubated with primary antibodies at 4 °C overnight. The embryos were washed in PBST for 5 min twice and then incubated with fluorochrome-conjugated secondary antibodies at room temperature for 1 hr. Nuclei were visualized by incubation with 2 μg/ml Hoechst 33342 for 10 min at room temperature. For the visualization of skin lymphatic endothelial cells, we followed the protocol by James *et al*.^37^. We used goat anti-Prox1 antibody (AF2727, R&D systems, Minneapolis, MN, USA).

### Confocal microscopy

Images of embryos were captured by a BioRevo inverted microscope with a GFP-B filter (Keyence Osaka, Japan) or Leica MZ FLIII with a GFP LP filter (Leica, Wetzlar, Germany). Confocal images were acquired on a Leica TCS-SP8 (Leica). Embryos were mounted in 80% glycerol on glass-bottom dishes (IWAKI, Tokyo, Japan). Fluorescence was excited with a 405-nm UV laser for Hoechst 33342, a 638-nm laser for Cy5 or Alexa Fluor 633, a 552-nm laser for Cy3, and a 488-nm laser for Alexa Fluor 488. A hybrid detector was used for signal amplification.

### Aortic ring assay

The aortic ring assay was performed as described previously^22^. Briefly, thoracic aortae were excised from *Flk1-Nano-lantern* BAC Tg mice, and peri-aortic tissues, such as the fat layer and adventitia, were removed using fine microdissecting forceps and iridectomy scissors. Rings of approximately 1 mm in length were prepared in serum-free Dulbecco’s modified Eagle’s medium (DMEM) (Sigma, St. Louis, MO, USA). Individual rings were embedded in type I collagen gel (Nitta Gelatin, Osaka, Japan) on glass-bottom dishes and cultured in DMEM containing 50 ng/ml human recombinant VEGF-A (R&D systems). After 1 week of culture at 37 °C in a humidified environment, the collagen gels containing the aortic rings were fixed in 4% paraformaldehyde for 30 min.

### Luminescence imaging of aortic ring assay

For luminescence imaging, 5 mM coelenterazine-h (Wako, Osaka, Japan) dissolved in EtOH was added to 2 ml of the medium (final concentration of 5 μM) in a glass-bottom dish. Half of the imaging media was replaced with media containing 5 μM coelenterazine-h every 6 hr. A LV200 inverted microscope with a 20×, NA 0.70, UCPlanFLN objective (Olympus, Tokyo, Japan) was used for the aortic ring assay. Luminescence was recorded by an ImagEM × 2 EMCCD camera (Hamamatsu, Shizuoka, Japan) without an emission filter every 30 min.

### Reverse-transcription (RT)-qPCR analysis

Total RNA was extracted using an RNeasy Mini kit (Qiagen, Hilden, Germany). For RT-qPCR analysis, first-strand cDNA was synthesized from total RNA using a QuantiTect Reverse Transcription kit (Qiagen). qPCR was performed with SYBR Premix Ex Taq II (Takara, Shiga, Japan) on a Thermal Cycler Dice Real Time System (TP850; Takara). The amount of target RNA was estimated using an appropriate standard curve and divided by the estimated amount of β-actin mRNA.

### Tumour implantation

Lewis lung carcinoma (LLC) cells were cultured in DMEM containing 10% foetal bovine serum (Sigma). After the LLC cells were trypsinized, 1 × 10^6^ cells were suspended in D-PBS(−) (Nacalai Tesque, Kyoto, Japan) and implanted into the back of a mouse. To examine tumour angiogenesis, we implanted LLC cells in 12-week-old WT and *Flk1-Nano-lantern* BAC Tg mice.

### Isolation of tumour ECs from mice

ECs from the skin or tumours in mice were isolated as previously described^38^. In brief, tumour or skin tissues were finely minced after resection and digested with dispase II (Godo Shusei, Chiba, Japan), collagenase type I (Wako, Osaka, Japan), and collagenase type II (Worthington Biochemical Corp., Lakewood, NJ, USA) in a 50-ml tube. The digested tissue was passed through 40-μm filters to yield single cell suspensions. Fluorescence-labelled anti-CD45 and -CD31 mAbs (BD Biosciences, San Jose, CA, USA) were used. CD31 + CD45-ECs were sorted by using a SORP FACSAria (BD Biosciences).

### Bioluminescence imaging of tumour angiogenesis

Video-rate imaging of freely moving *Flk1-Nano-lantern* BAC Tg:: B6 (Cg)-Tyr^c−2J^ mice and WT B6 (Cg)-Tyr^c−2J^ mice was performed as described previously19. Briefly, 8 g of coelenterazine-h (12 μl EtOH + 48 μl D-PBS(−); final volume 60 μl) was injected inside the LLC tumour using an insulin syringe with a 23 G needle followed by imaging after 1 min. For video-rate observation, we used a Lumazone *in vivo* luminescence imaging system (Molecular Devices, California, USA) equipped with an Evolve512 EMCCD camera (Photometrics, Arizona, USA) and a HF12.5SA-1 lens (Fujifilm, Tokyo, Japan). Bright field images under illumination by a LightEngine SPECTRA (Lumencor, Oregon, USA) and luminescence images taken in the dark were captured alternatively. To adjust the timing of the light illumination, pulsed signals for turning on the light were generated by a WF1973 multifunction generator (NF Corporation, Yokohama, Japan) according to exposure time-out signals from the EMCCD camera.

### Flow cytometry

Flow cytometric analysis was performed as described previously. Fluorescent dye-labelled anti-Flk1 (BD Biosciences, San Jose, CA, USA, clone AVAS12) was used to stain the cells. Stained cells were analysed with a FACSCalibur (BD Biosciences). Data were analysed by FlowJo software (TreeStar, Ashland, OR, USA).

### Statistical analysis

Student’s *t* tests were used to assess the level of significance between groups.

## Additional Information

**How to cite this article:** Matsushita, J. *et al*. Fluorescence and Bioluminescence Imaging of Angiogenesis in *Flk1-Nano-lantern* Transgenic Mice. *Sci. Rep.* **7**, 46597; doi: 10.1038/srep46597 (2017).

**Publisher's note:** Springer Nature remains neutral with regard to jurisdictional claims in published maps and institutional affiliations.

## Supplementary Material

Supplementary movie 1

Supplementary movie 2

Supplementary Figs and Tables
