# Supplementary material for: Fluorescence and Bioluminescence Imaging of Angiogenesis in Flk1-Nano-lantern Transgenic Mice
Source: Sci Rep. 2017 Apr 20;7:46597. doi: 10.1038/srep46597 (PMC5397864; doi:10.1038/srep46597)
Supplement: Supplementary Figs and Tables [file srep46597-s3.pdf]

Supplementary Information

Fluorescence and Bioluminescence Imaging of Angiogenesis in *Flk1-Nano-lantern* Transgenic Mice

Jun Matsushita[1,¶], Shigenori Inagaki[2,¶], Tomomi Nishie[1], Tomoki Sakasai[3], Junko Tanaka[3], Chisato Watanabe[4], Ken-ichi Mizutani[4], Yoshihiro Miwa[3], Ken Matsumoto[1], Kazuhiro Takara[5], Hisamichi Naito[5], Hiroyasu Kidoya[5], Nobuyuki Takakura[5], Takeharu Nagai[2], Satoru Takahashi[6,7,8], Masatsugu Ema[1,9]*

Figure Legends

Supplementary Figure 1. Expression of Venus in *Flk1-Nano-lantern* BAC Tg mice

(A) Gross appearance of *Flk1-Nano-lantern* BAC Tg mice (#2). Bright field and Venus fluorescence images of newborns are shown. (B) Fluorescence of *Flk1+/GFP* and *Flk1-Nano-lantern* BAC Tg embryos at E9.5. Scale bars: 500 μm (C) Venus expression in blood vessels of a 9.5-dpc *Flk1-Nano-lantern* BAC Tg embryo. Arrows indicate ISV. ISV: intersomitic vessel. Scale bars: 200 μm.

Supplementary Figure 2. Co-expression of endogenous Flk1 and *Nano-lantern* driven by the *Flk1* regulatory sequence in vascular ECs

Co-expression of Venous and endogenous Flk1 protein in *Flk1-Nano-lantern* BAC Tg embryos (#2) at 9.5 dpc. Tg embryos were subjected to immunohistochemical analysis with anti-GFP and anti-Flk1 antibodies. * indicates strong autofluorescence caused by circulating blood cells. Circulating blood cells localised in blood vessels are also shown by arrows. Open arrowheads and closed arrowhead indicate intersomitic vessels and vitelline vein, respectively. Scale bars: 100 μm.

Supplementary Figure 3. Expression of *Flk1-Nano-lantern* in lymphatic ECs

Immunohistochemical analysis of *Flk1-Nano-lantern* BAC Tg mice with anti-GFP, -Flk1 and -Lyve1 antibodies. Scale bars: 100 μm. Closed and open arrowheads indicate Lyve1-positive lymphatic ECs and Lyve1-negative vascular ECs, respectively. Arrows indicate Lyve1-positive macrophages. Scale bars: 100 μm

Supplementary Figure 4. Expression of Venus in aortic ECs of *Flk1-Nano-lantern* BAC Tg mice

Venus expression in aortic rings from *Flk1+/GFP* mice and *Flk1-Nano-lantern* BAC mice (#2 and #23). Aortic rings were isolated from *Flk1+/GFP* mice and *Flk1-Nano-lantern* BAC Tg mice (#2 and #23), and then cultured in the presence of VEGF-A. Endothelial sprouts were fixed and immunostained with anti-GFP and anti-Flk1 antibodies. Scale bars: 100 μm.

Supplementary Movie S1. Luminescence imaging of ECs in aortic rings from Flk1-Nano-lantern BAC Tg mice

The observation of EC sprouting was achieved by capturing the luminescence every 30 min over a period of 18 hr.

Supplementary Movie S2. Bioluminescence imaging of tumour blood vessels in Flk1-Nano-lantern BAC Tg mice

Luminescence from the tumour ECs in freely moving mice was observed at a video-rate.

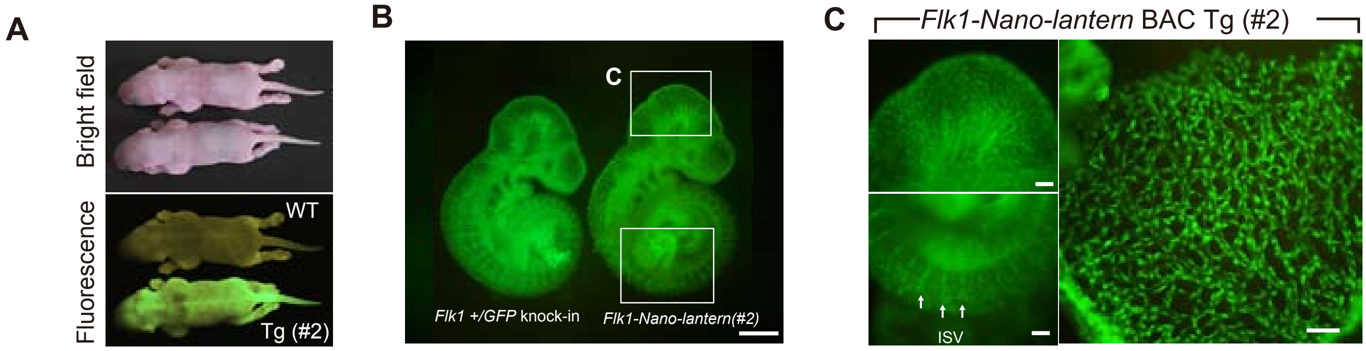

Matsushita et al., Supplementary Figure 1

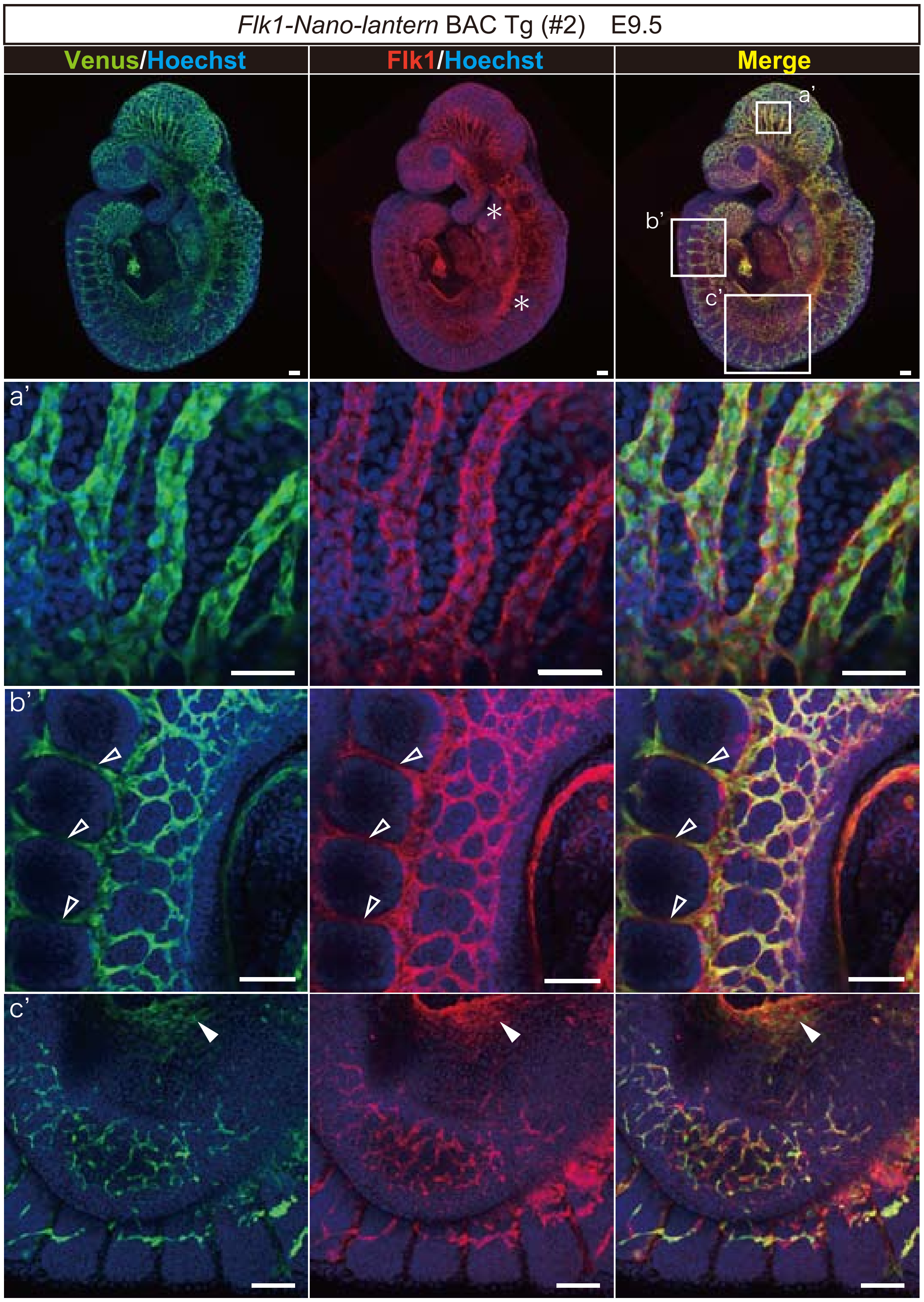

Matsushita et al., Supplementary Figure 2

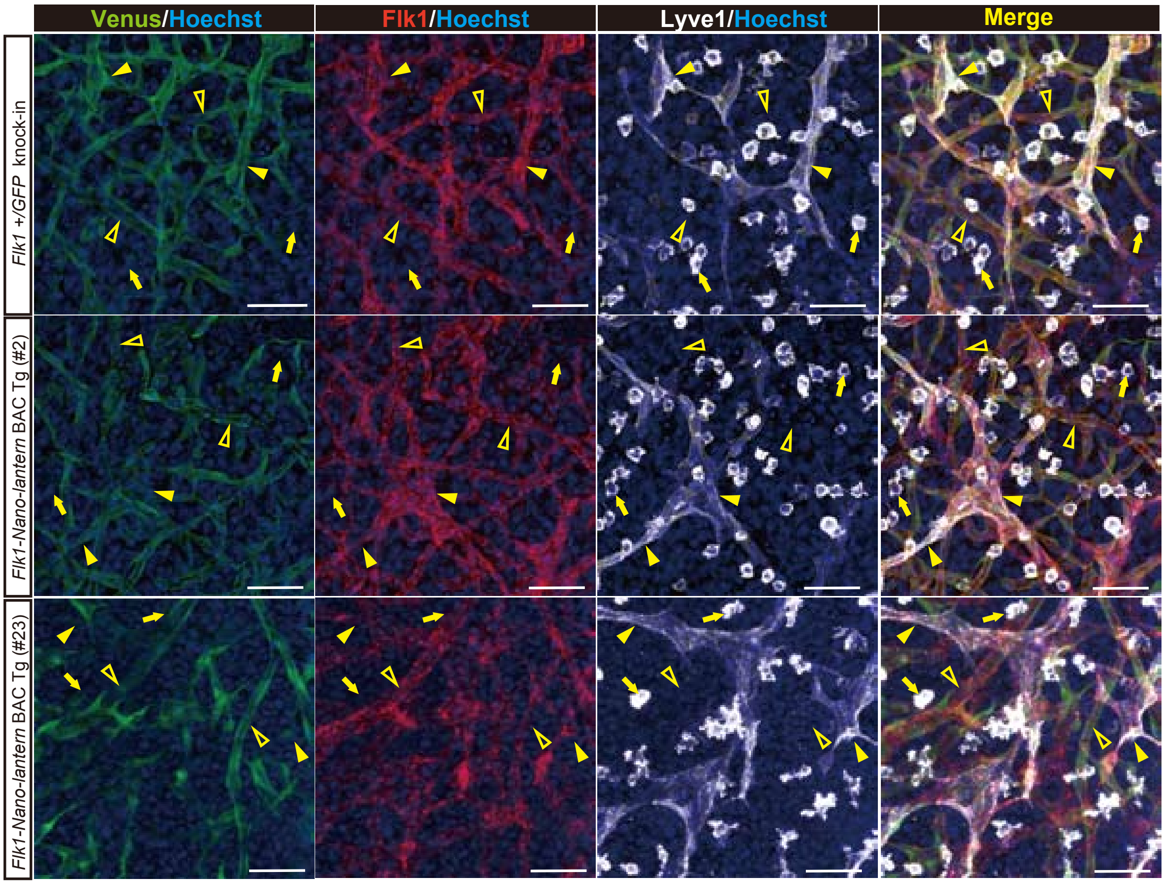

Matsushita et al., Supplementary Figure 3

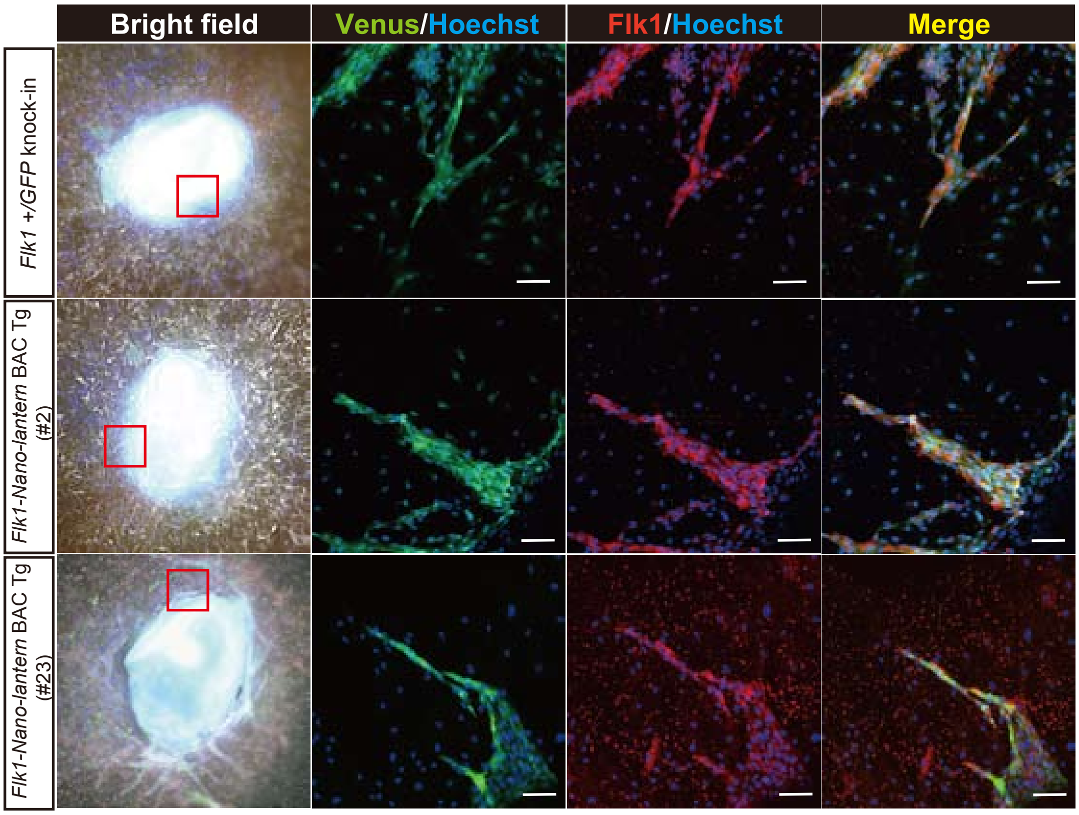

Matsushita et al., Supplementary Figure 4

Table S1: List of RT-qPCR primer sequences

Gene	Primer Sequence (Forward)	Primer Sequence (Reverse)
β-actin	GTGTAAAACGCAGCTCAGTAACAGT	CTGAGCGCAAGTACTCTGTGTG
Flk1	GGGATGGTCCTTGCATCAGAA	ACTGGTAGCCACTGGTTG
PECAM1	ATCCGGAAGGTCGACCCTAATCTCAT	ATACCCAACATGAACAAGGCAGCG
VE-cadherin	GGATGCAGAGGCTCACAGAG	CTGGCGGTTCACGTTGGACT
